# A review of clinical photoacoustic imaging: Current and future trends

**DOI:** 10.1016/j.pacs.2019.100144

**Published:** 2019-11-07

**Authors:** Amalina Binte Ebrahim Attia, Ghayathri Balasundaram, Mohesh Moothanchery, U.S. Dinish, Renzhe Bi, Vasilis Ntziachristos, Malini Olivo

**Affiliations:** a Laboratory of Bio-optical Imaging, Singapore Bioimaging Consortium, A*STAR, Singapore; b Institute for Biological and Medical Imaging, Technische Universität München and Helmholtz Zentrum München, Ingolstädter Landstraße 1, 85764 Neuherberg, Germany

**Keywords:** AR-PAM, acoustic resolution-photoacoustic microscopy, DAQ, data acquisition, FOV, field-of-view, HbO_2_, oxy-hemoglobin, Hb, deoxy-hemoglobin, LED, light emitting diode, MAP, maximum amplitude projection, MEMS, microelectromechanical systems, MRI, magnetic resonance imaging, MSOT, multispectral optoacoustic tomography, OCT, optical coherence tomography, OR-PAM, optical resolution-photoacoustic microscopy, RSOM, raster-scanning optoacoustic mesoscopy, PA, photoacoustic, PAI, photoacoustic imaging, PAM, photoacoustic microscopy, PAT, photoacoustic tomography, SBH-PACT, single breath hold photoacoustic computed tomography system, sO_2_, saturation, US, ultrasound, Photoacoustic imaging, Optoacoustic tomography, Optoacoustic mesoscopy, Photoacoustic microscopy, Clinical applications

## Abstract

Photoacoustic imaging (or optoacoustic imaging) is an upcoming biomedical imaging modality availing the benefits of optical resolution and acoustic depth of penetration. With its capacity to offer structural, functional, molecular and kinetic information making use of either endogenous contrast agents like hemoglobin, lipid, melanin and water or a variety of exogenous contrast agents or both, PAI has demonstrated promising potential in a wide range of preclinical and clinical applications. This review provides an overview of the rapidly expanding clinical applications of photoacoustic imaging including breast imaging, dermatologic imaging, vascular imaging, carotid artery imaging, musculoskeletal imaging, gastrointestinal imaging and adipose tissue imaging and the future directives utilizing different configurations of photoacoustic imaging. Particular emphasis is placed on investigations performed on human or human specimens.

## 1 Introduction

Photoacoustic (PA) imaging (PAI), or optoacoustic imaging, is a hybrid imaging modality that merges optical illumination and ultrasound (US) detection [1,2]. It has been rapidly gaining popularity and explored for biomedical imaging applications in the last two decades. As pure optical imaging methods cannot maintain high-resolution imaging in deep biological tissues due to optical scattering, the capability to achieve high resolution optical contrast images in biological tissues up to centimeters depths makes PAI a promising technique for various clinical imaging applications. In PAI, a nano-second pulsed laser (pulse duration < 10 ns) is commonly used to illuminate the biological sample. The molecules absorb the optical energy and convert it into heat, generating a temperature rise. The thermoelastic expansion from the temperature rise generates acoustic waves which is detected using ultrasonic transducers. As sound scatters 1000 times lesser than light [3,4], the acoustic signal propagates much longer in biological tissue without significant attenuation.

PAI capitalizes mainly on the intrinsic optical absorption of chromophores in human tissue such as hemoglobin, melanin, lipid and water. As each of these chromophores exhibits its own characteristic absorption spectra, PAI at multiple wavelengths allows their relative quantification and helps to investigate physiological changes in disorders to understand the mechanism behind them and how they can be managed effectively. Due to its label-free nature, it encourages patients’ compliance and is capable of long-term longitudinal monitoring. Because PAI can differentiate between deoxy-hemoglobin (Hb) and oxy-hemoglobin (HbO_2_), measuring the sO_2_ in the blood vessels is possible, which is a significant characteristic of ischemia, hypoxia or hypoxemia physiological states for other applications [5]. PAI can exploit the optical absorber in skin, melanin, for imaging of melanoma, pigmented lesions and hair follicles. The presence of lipids in certain areas such as arterial plaques and sebaceous glands makes it convenient for PAI interrogation. However, if the endogenous contrast is not sufficient due to the similarity of absorption spectra of one or more chromophores, various exogenous contrast agents can also be employed to augment PAI for better contrast and deeper imaging [[6], [7], [8]]. It is interesting to note that clinically relevant FDA approved contrast agents such as indocyanine green, ICG [9] and methylene blue [10] are photoacoustically strong chromophores.

**Fig. 1:**
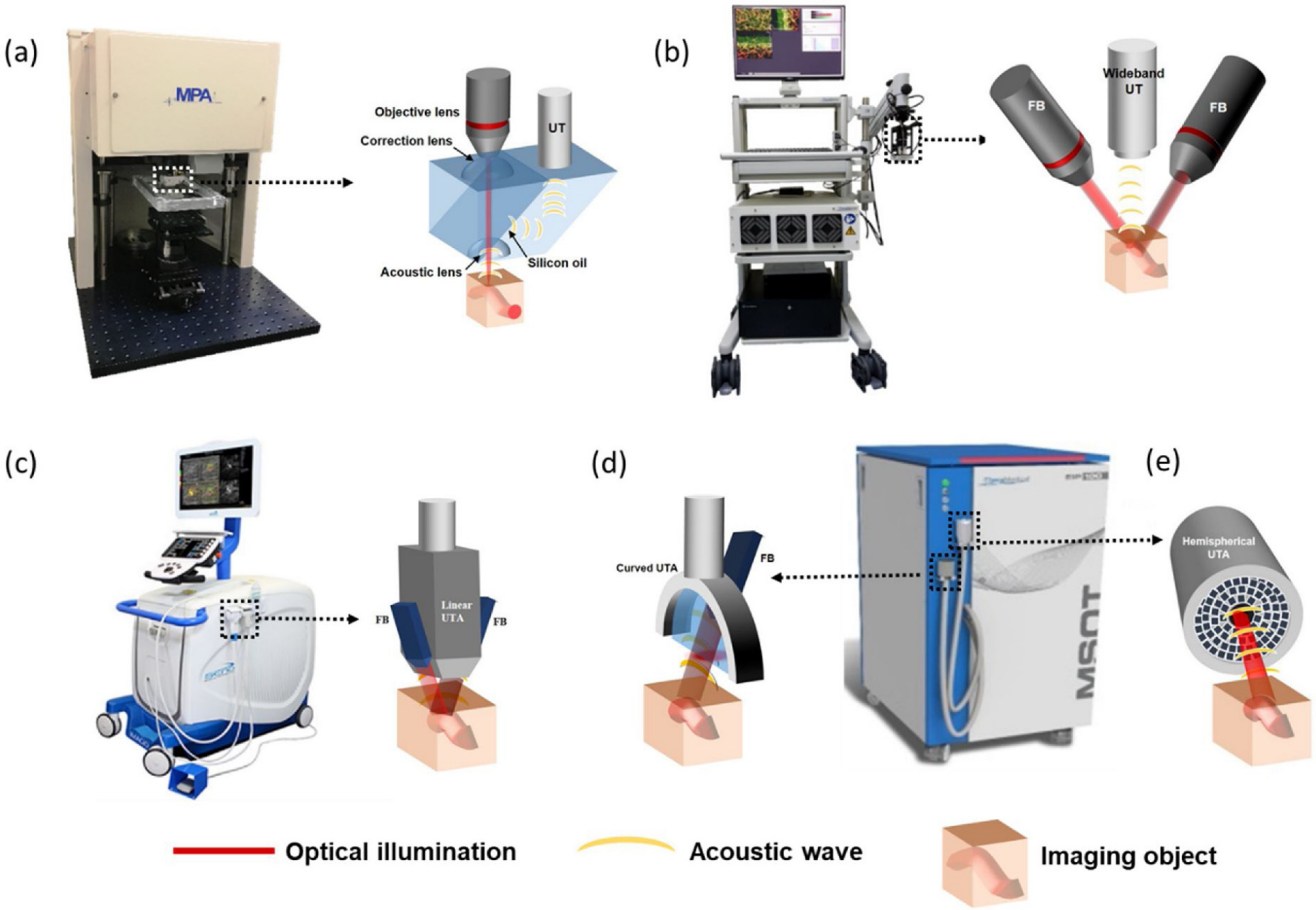
Schematic of different modes of photoacoustic imaging system along with the commercial systems used for clinical applications. (a) Optical resolution photoacoustic microscopy (OR-PAM) (b) Raster-scanning optoacoustic mesoscopy (RSOM) and photoacoustic tomography (PAT) equipped with (c) linear, (d) curved, (e) spherical transducer array in hand-held mode. FB, fiber bundle; UTA, ultrasound transducer array; UT, ultrasound transducer.

Generally, PAI systems can be grouped into three configurations subjected to their combination of optical illumination methods and acoustic detection methods: tomography, mesoscopy and microscopy systems (Fig. 1) [11]. A typical photoacoustic tomography (PAT) system uses wide-field illumination and detects the acoustic waves by a single element [12,13] or array transducer [14,15]. The use of a single element transducer is time-intensive and affords only static imaging, making it inadequate for *in vivo* clinical imaging applications. By using multiple transducers for detection, the emitted acoustic waves can be captured at multiple angles and hence ideal for real time monitoring of the imaging region with increased acquisition speed. As the transducers can be arranged in 2D or 3D spatially, a 2D cross-sectional or 3D volumetric image can be reconstructed from all projections. Depending on the applications and imaging targets, the transducer array can be mounted on a stationary platform [16,17] or be used in hand-held imaging mode [18,19]. The latter imaging configuration is especially practical in clinical practice for better accessibility to the various parts of the human body. The 2D array transducer can be either linear or curved. Linear array based PA systems can be developed using commercial ultrasound machines [[20], [21], [22]]. They are however hampered in detecting acoustic signals at limited angles from the region of interest as compared to curved transducer array systems [17]. If the transducers can be arranged spatially in 3D, acoustic waves can be detected from all in plane directions and hence complete volumetric images can be acquired in a single frame. A laboratory prototype of 3D array transducers with wide field-of-view (FOV) [23,24] and commercial handheld transducer with small FOV [25] can be used for various clinical imaging needs. Several companies, including iThera Medical GmbH, FUJIFILM VisualSonics, TomoWave Laboratories Inc., ENDRA Life Sciences Inc., Canon and Seno Medical Instruments, etc. provide commercial transducer-array-based PAT systems for medical imaging. Even though PAT systems are able to visualize vascular structures and intrinsic contrast at centimeters depth, they are not well-suited for high-resolution superficial vascular imaging applications.

Photoacoustic microscopy (PAM) on the other hand is a high-resolution PAI technique which can further be categorized into two implementation methods: acoustic resolution-PAM (AR-PAM) and optical resolution-PAM (OR-PAM) [26]. Deep tissue imaging at high resolution (∼ 20−50 μm) beyond optical diffusion limit (∼ 1 mm in biological tissue) can be accomplished in AR-PAM by utilising weak optical and tight acoustic focusing. The lateral resolution of AR-PAM is dependent on the centre frequency of the ultrasound transducer and the acoustic lens used, with the most common configuration being 50 MHz ultrasound transducer and 0.44 N.A acoustic lens to give a lateral resolution of ∼45 μm and penetration depth of up to 3 mm [27,28]. In order to achieve high lateral resolution down to single capillary (∼ 5 μm) using AR-PAM, high-frequency transducers are needed (higher than 400 MHz central frequency), which restricts the penetration depth to less than 100 μm. Instead, the lateral resolution can be enhanced by a tight focusing of an optical beam as in an OR-PAM setup [29,30], which enables organelle and cellular level imaging from its high lateral resolution spanning hundreds of nanometer to several micrometers [31]. In an OR-PAM setup, a spatially filtered laser beam is focused using a microscope objective to pass through an optoacoustic beam combiner comprising of both right angled and rhomboid prisms with a coating of silicon oil in between which functions as an optically transparent and acoustically reﬂective ﬁlm. An acoustic lens below the rhomboid prism provides acoustic focusing. An ultrasonic transducer placed on top of the rhomboid receives the acoustic signals. For OR- and AR-PAM image acquisition, two-dimensional continuous raster scanning imaging heads are used. An A-line is the time-resolved PA signals multiplied by the speed of sound, 1540 m/s in soft tissue. Multiple A-lines acquired during the continuous Y stage movement produces a two-dimensional B-scan. Multiple B-scans are acquired and processed to obtain the maximum amplitude projection (MAP) photoacoustic images. Recently, the use of microelectromechanical systems (MEMS) has improved OR-PAM imaging speed significantly [32,33]. The implementation of MEMS in the setup will help in the miniaturization of the technique and will be highly beneficial for the development of endoscopic devices and in clinical translation.

iThera Medical GmbH, modified the concept of AR-PAM by using a customized ultra-broadband transducer and a two-arm fiber bundle light delivery, with this specific implementation termed as photoacoustic mesoscopy or raster-scanning optoacoustic mesoscopy (RSOM). Herein, wide field optical illumination and a single tightly focused high center frequency (typically ≥ 50 MHz) US transducer with a wide bandwidth range (10–180 MHz) are utilized to achieve high spatial resolution, (typically 15–40 μm) [34]. By reconstructing the acquired data and segmenting them into two different frequency bands, e.g. 10–30 MHz (low) and 30–90 MHz (high), and then spatially merging them together, the location of capillaries and bigger blood vessels can be differentiated. Achieving a depth of 2 mm, this configuration has been demonstrated to be highly useful in understanding conditions related to human skin like psoriasis [34] and eczema (unpublished data).

As such, all three configurations of PAI systems mentioned above have demonstrated unparalleled advantages in certain clinical applications, including deeper imaging of biological tissue, high spatial resolution for *in vivo* imaging, contrast-free and stain-free imaging capability of endogenous molecules which are discussed in detail below.

## 2 Clinical applications of photoacoustic imaging

### 2.1 Breast imaging

Photoacoustic imaging of the breast has garnered immense interest among researchers worldwide owing to the penetration depth it can comfortably achieve to cover the entire, if not, most of the breasts. Breast cancer is the most commonly occurring cancer in women with over 2 million new cases in 2018 [35]. Imaging plays a significant role in breast screening, diagnosis, staging and monitoring therapeutic interventions. While X-ray mammogram is the most commonly used method to screen and diagnose breast cancer in spite of it being painful, having the ill-effects of exposure to ionizing radiations and reduced accuracy in denser breast [36], ultrasonography follows next, especially to differentiate between benign and malignant cysts [37] with magnetic resonance imaging (MRI) and other nuclear imaging modalities [38] prescribed to lesser extent. Angiogenesis and hypoxia being few of the major hallmarks of cancer, sensitivity of optical imaging to the different forms of hemoglobin make it highly reliable over other imaging modalities and encourage using it in combination with other radiology methods [39] to improve the sensitivity. However, because of the penetration limit of light to ∼1 mm in tissues, the clinical translation of optical methods for breast cancer have not been successful. PAI effectively breaks through this penetration depth by combining functional imaging and contrast of optical tomography and high spatial resolution of US to reach depths of up to 4 cm [23] and thus gathering immense interests from many research groups, particularly for breast cancer as it is one of the major superficial organs. Several configurations of the photoacoustic systems have been and are being developed by various groups to obtain the maximum possible information from breasts of various phenotypes to give high spatial and temporal resolution images of angiographic structures with minimal motion artifacts. Different *in vivo* studies performed on normal and breast cancer patients and *ex vivo* studies performed on breast specimen will be discussed briefly in this section.

#### 2.1.1 *In vivo* imaging

The first use of PA for imaging breasts in patients was demonstrated as early as 2001 by Oraevsky et al. [40]. Following this, several studies demonstrating the technical utility of various manifestations of PA breast imaging systems to visualize malignancies in human breasts were published [[41], [42], [43], [44], [45], [46]]. One of the first clinical studies performed by Manohar et al. was using a stand-alone clinical breast imaging system called the Twente Photoacoustic Mammoscope which uses a single NIR laser, 1064 nm for excitation and an ultrasound detector array with 588 elements providing illumination from the cranial direction. The system has metamorphosed through a few versions to acquire PA signals from a field of view (FOV) of 9 × 8 cm^2^ within 10 min with a resolution of approximately 3.5 mm [47]. With the PA images obtained using this system, 32 of the 33 lesions could be detected successfully [24]. Moreover, the PA appearances of these high intensity lesions which can be broadly categorized into – 1) mass-like, 2) ring-like and 3) non-mass or scattered were similar to those observed on MR dynamic contrast enhanced (DCE) imaging and vascular stained histopathology of breast malignancies suggesting the contribution of vasculature to the high intensity lesions spotted in malignancies than lipids or collagen which could also contribute to PA contrast [47]. Most importantly, the contrast of the lesion was independent of the breast density [24], which when higher makes the diagnosis of tumor nearly impossible on X-Ray mammogram [41]. The problem of imaging denser breasts has been demonstrated to be overcome by also using a volumetric three dimensional photoacoustic handheld probe consisting of a spherical matrix array of 256 ultrasound sensors each with a central frequency of 4 MHz [48]. In healthy volunteers of ages 25 and 40 whereby the breast tissue is known to contain dense fibroglandular tissue that will make tumor diagnosis difficult on X-ray mammogram, Deán-Ben et al. have shown volumetric views of the breast vasculature and surrounding parenchyma at a single wavelength (800 nm) excitation up to a depth of 1 cm. In addition, by using multiple wavelengths, such as 730, 760 and 850 nm from a tunable optical parametric oscillator (OPO) laser for tissue excitation, they have offered high resolution volumetric maps of blood oxygenation parameters like HbO_2_, Hb and total hemoglobin (HbT) [49]. By offering PA images at a high resolution of 200 μm, this study is the first of its kind to help look for angiogenic biomarkers and regions with high microvessel density which are the characteristics of breast cancer growth and invasion [50,51].

**Fig. 2:**
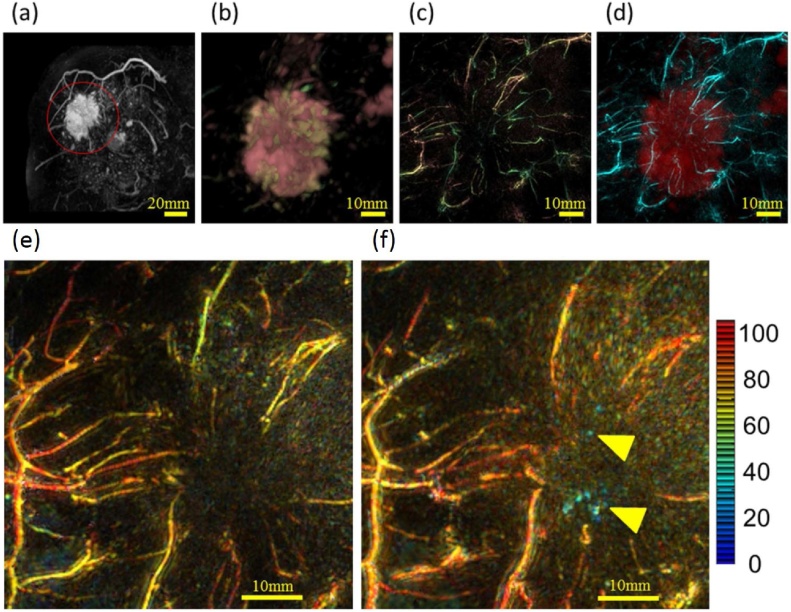
PA mammoscope images of peritumoral vasculature in IBC of a 40-year-old woman. (a) Original MR image with lesion (47 mm diameter) indicated by red circle, (b) enlarged MR image around the lesion after deforming into the shape to PA image, (c) original PA image, (d) fused PA (cyan) and MR (red) image, (e) enlarged PA image at 795 nm (signifying hemoglobin distribution) showing lesser intratumoral vessels before chemotherapy and (f) enlarged PA image at 795 nm showing increased number of finer intratumoral vessels after chemotherapy. Relative sO_2_ values indicated according to the color bar show a decrease in (f) compared to (e) indicating hypoxia (yellow arrows). Reprinted with modification from Ref. [52], image licensed under http://creativecommons.org/licenses/by/4.0/.

Interestingly, PAI’s capacity to derive oxygenation status of blood vessels has been demonstrated to aid largely in assessing anti-cancer treatment driven changes in the blood vessels [52]. Following chemotherapy with taxane, the relative oxygen saturation in peritumoral blood vessels was observed to be low indicating hypoxia (Fig. 2). At the same time, finer intratumoral blood vessels became noticeable suggesting increased blood perfusion possibly due to the chemotherapy associated lowering of interstitial blood pressure leading to normalization of blood vessels. Apparently, all these finer changes happened without any change in the tumor size as observed by ultrasound, thus reinstating the potential advantages that PAI can bring to the clinics. In the same study, remarkable differences were noticed in the shape and the number of blood vessels between the diseased and contralateral breast, opening the avenue for analysis of finer blood vessels to diagnose early breast cancer. Moreover, the observation of centralizing vasculature signal tending towards the tumor in 61 % of invasive breast carcinoma (IBC) cases as opposed to only 35 % of ductal carcinoma in situ (DCIS) cases may eventually help in distinguishing IBC from DCIS lesions. The authors were able to provide more comprehensive vascular structural features with depth profile unlike previous studies mainly by exploiting the technological advances. The authors addressed the well-documented drawbacks of limited view associated with planar configuration of transducer arrays [11] by using hemispherical array and applying the breast misshapement algorithm from the MR breast shape image to that in a PA image, from which functional information about peritumoral and intratumoral vasculatures were obtained. In addition there have been a few multicenter studies with the first CE marked photoacoustic breast imaging system, Imagio by Seno Medical Instruments demonstrating the ability of ultrasound guided photoacoustic imaging to rightly diagnose benign and malignant breast masses [53] based on the relative amount of HbO_2_ and Hb and further downgrade benign masses classified as Breast Imaging Reporting and Data System (BI-RADS) 4a and 4b to BI-RADS 3 and 2 thus potentially decreasing benign breast biopsies [54].

The capacity of PAI to distinguish between not just the different forms of hemoglobin but between many intrinsic chromophores like melanin, lipids and water was realized with the introduction of fast tunable OPO lasers [55]. As human breasts are composed mainly of vasculatures, fats and water, it is of benefit not just to look at the angiography of the breast but also to look at the distribution of fats within the breasts and in the tumors. In a pilot study, Diot et al. used a handheld photoacoustic probe with extended spectral range spanning 28 wavelengths from 700 nm to 970 nm to compute the contributions of Hb and HbO_2_, total blood volume (TBV), lipid and water and to derive multispectral optoacoustic tomography (MSOT) patterns in normal and cancerous breasts of various stages [56]. Angiogenesis, the characteristic hallmark of cancer was visualized as increased peripheral vascularization on Hb, HbO_2_ and TBV images and decreased intratumoral vascularity, mainly because the intratumoral vessels were too small to be detected by the transducer. Tumor areas appeared to have heterogenous TBV patterns with rim measuring more than 30-fold compared to the neighboring non-cancerous regions and presented as disruptions in fat and water layers in the place of tumor as the tumors were replacing the original fat and water layers of the breast. Additionally, skin infiltration of breast tumor presented itself as a disruption of melanin and skin vascularization. Understanding these breast cancer features of MSOT could potentially guide surgery and overall therapy planning.

In spite of the compelling demonstrations of clinical utility of PAI in breast cancer as discussed above, there are several avenues for development addressing which the clinical applicability can be actualized. For instance, PAI has been demonstrated to be sensitive to hormone-related physiological changes of breast parenchyma [57] like MRI [58], elastography [59] and near-infrared spectroscopy measurements [60] which will likely have an impact on the contrast of the images. By understanding and characterizing the normal ranges of breast vascularity parameters in pre- and post-menopausal women and at different phases of menstrual cycle, quantitative cut-offs for differentiating between benign and malignant lesions could be established. Also, as breast imaging is largely affected by motion artefacts arising from the movements of heart and lungs, continued efforts are taken to correct for breathing motion through post processing algorithms [52]. As such, shorter imaging times of the scanner will be an added advantage. When most of the mammoscopes described above take a few minutes for imaging which itself is good enough to induce motion artefacts, a single breath hold photoacoustic computed tomography system (SBH-PACT) developed by Lin et al. [23] will be a substantial alternative to address this. SBH-PACT could reveal the angiographic morphology from the nipple to the chest wall in a healthy 27 year old woman in a single breath hold’s time of 15 s. Addressing the limited view drawback [11] by using ring detectors that can be switched between 2D and 3D modes, eight out of nine breast cancer lesions were accurately detected using high spatial (255 μm) and temporal resolution images. However, in order to keep the acquisition time short, single wavelength imaging (1064 nm) was used, making it difficult to obtain oxygenation information of tumors.

#### 2.1.2 *Ex vivo* imaging

For early stage breast cancers, breast conserving surgery (BCS) or lumpectomy in conjunction with radiation therapy is emerging as a popular and efficient alternative to total mastectomy [61]. However, 20–70% of BCS patients undergo more than one surgical procedure before achieving tumor free margins [62,63]. Current standard of care for verifying tumor free margins includes histopathology and frozen sections [64,65] which suffer from long procedure times, on-table ultrasound [66] which suffers from operator dependent variability and x-ray mammogram [67], which has shorter processing time but reduced sensitivity and specificity due to poor contrast in denser breast tissues as mentioned earlier and the lack of functional specificity. When optical technologies like diffuse reflectance spectroscopy [68], optical coherence tomography [69] and Raman spectroscopy [70] are contributing to improve the sensitivity and specificity of tumor margin detection, they are still limited by long processing times, limited penetration depth and FOV. PAI with proven capability in rapid-deep tissue biochemical imaging [55] especially for *in vivo* breast cancer imaging (as discussed earlier) is emerging as a technique of immense interest for *ex vivo* breast specimen analysis too, particularly for assessing the margins. Interestingly, all clinical studies in this space have used ultrasound guided photoacoustic tomography systems.

In one of the pioneering studies, Li et al. used multiple wavelengths spanning from 1100 to 1250 nm in the second optical window at a step size of 10 nm to excite the excised breast specimen fixed in 10 % buffered formalin and obtained the distribution of hemoglobin and fat [71]. By classifying fatty tissue with scattered connective tissue as normal tissue and the rest as cancerous, assessment of 12 specimens fixed in 10 % buffered formalin using PAI resulted in 100 % sensitivity and 75 % specificity in assessing the tumor margins. The reduced specificity was mainly attributed to the dense connective tissue being picked up as cancer. This could be improved by using strict classifications to identify the two which could be achieved by looking further into their mechanical properties through frequency differences between the two. As an extension of this concept, Li et al. engineered an intraoperative multimodal ultrasound and PAT system that made use of overtone absorption of fat at 1197 nm as contrast [72]. The usage of single wavelength greatly reduced the scan time to 2 min covering an area of 10 × 10 cm^2^ breast specimen. In a clinical study involving breast cancer specimen from 66 patients, this system was observed to assess tumor margins with a sensitivity of 85.5 % and specificity of 90 % through machine learning algorithms.

In a pilot exploratory study performed by Goh et al., they made use of the differences in the absorption of hemoglobin and fat in the wavelengths ranging from 700−1100 nm to image their distribution in freshly excised breast specimens [17]. Tumors being hallmarked by angiogenesis, intense Hb signals were observed on all their edges. Samples with negative margins were noted to have continuous intense lipid layers on all edges of the tumor and those with positive margins were noted to have discontinuous lipid layers as observed in the *in vivo* PAI imaging of breast cancer by Diot et al. [56]. Based on these classifications, an assessment of 15 fresh specimens containing 90 margins in total (6 margins per sample) revealed a sensitivity of 100 % and a specificity of 97.6 % (in press). The reduced specificity was attributed to two of the negative margins being picked up as positive margins. Interestingly, the two incorrectly identified margins had close margins of < 0.5 mm as identified by histology. As no tumor on ink for invasive ductal carcinoma (IDC) and 2 mm for DCIS are the acceptable surgical margins according to the Society of Surgical Oncology [73,74], it can be considered that PAI was observed to have 100 % specificity as well.

Alternatively, an OR-PAM system operating with a laser in the UV range (266 nm), termed UV-PAM, has been demonstrated to offer histology-like imaging of fixed but unprocessed human breast specimen [75]. Diagnostic criteria such as nuclear size, internuclear distance and packing density make UV-PAM very promising in the detection of cancer cell clusters at the cancer specimen margins. UV-PAM in the published version would take 7 h for imaging 1 cm x 1 cm at a spatial resolution comparable to high-NA optical microscopes. However, by improving the optics in UV-PAM, it is possible to scan a BCS specimen with an average size of 5 cm diameter in 4 min.

### 2.2 Dermatologic imaging

The human skin, being easily accessible, makes it an ideal location for non-invasive imaging. Skin disorders are usually diagnosed by visual inspection and occasionally confirmed by skin biopsies and following histopathology. While this method remains the gold standard for skin disorder diagnosis, they are invasive and unaesthetically pleasing, especially on easily scarred Asian skin. Non-invasive imaging can potentially negate the need for skin biopsies with faster diagnosis times, and can benefit the treatment management of the disorder. However, the skin architecture is complex and can be regarded as a multilayer optical medium and this should be reflected and captured by these imaging modalities. Photoacoustic imaging in the mesoscopy scale allows for the different layers of the skin to be discerned [73]. RSOM imaging of a volunteer’s palm revealed the different skin layers, the uppermost layer making up epidermis of about 200 μm thickness followed by the epidermis-dermis junction [76]. It is also capable of visualizing vessels from capillary loops near the epidermis-dermis junctions, down to the arterioles and venules in the vascular plexus as described above. Additionally, the spectral capabilities conferred by MSOM imaging can differentiate between melanin, HbO_2_ and Hb [77]. The absorption maps of these tissue components can be reconstructed in volumetric spatial maps with the superficial epidermal layer distinguished by strong absorption of melanin and the vascular plexus underneath consisting of deoxy- and oxy-hemoglobin maps. The sO_2_ level of the vascular network was calculated at different depths and found to be 85 ± 4 % at the skin surface, and 54 ± 7 % of a deeper vein in the dermis. One limitation of RSOM/MSOM imaging is that it is sensitive to the subject’s motion (e.g. breathing) due to the focality of the ultrasound detector relative to the subject’s skin. To overcome this, a motion correction algorithm was reported that first depended on the segmentation of the skin's melanin [78] which subsequently evolved into an automatic algorithm by taking into account the vertical shifts of the skin at various body regions [79]. While the RSOM/MSOM and PAM systems allow for high-resolution visualization of vessels abnormalities; a common indicator for skin diseases; tomographic PAI systems will be more useful for larger and deeper skin structures such as the delineation of skin cancer.

#### 2.2.1 Pilosebaceous units

**Fig. 3:**
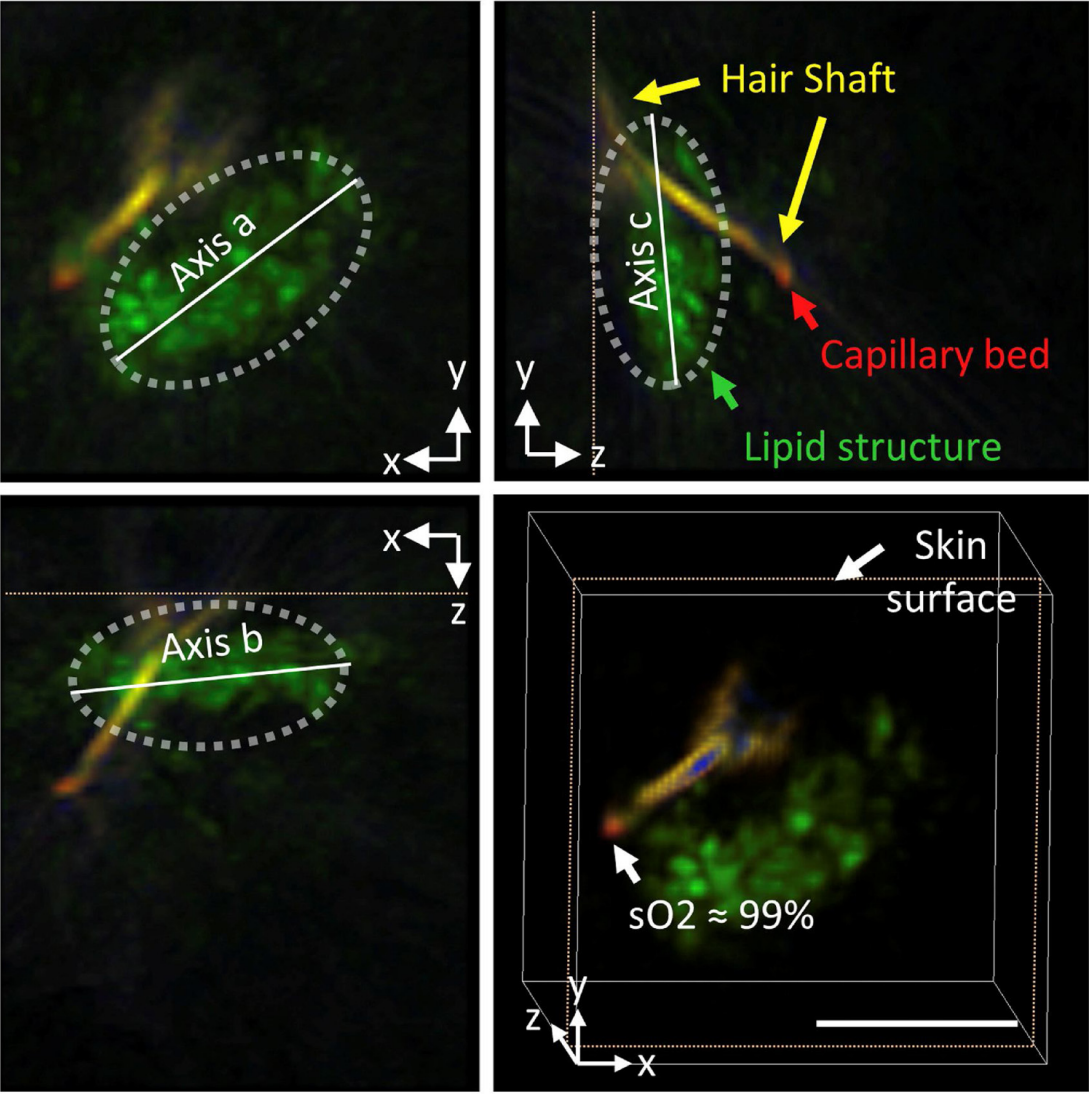
PA orthogonal images and 3D projection of a single pilosebaceous unit imaged on the forehead. Unmixed PAI signals show the spatial maps of HbO_2_ (red), Hb (blue), lipid (green) and melanin (yellow), revealing an ellipsoid-like lipid structure near the hair shaft. Notably, the sO_2_ of the capillary bed feeding the follicle was highly oxygenated. Scale bar; 3 mm. Reprinted with permission from Ref. [80].

MSOT has been exploited for the imaging of pilosebaceous units whereby the hair follicle structure was elucidated including the hair shaft, the dermal papilla and the sebaceous glands in the vicinity as seen in Fig. 3 [80]. By imaging at different wavelengths, the sO_2_ levels of the capillary bed encompassing the dermal papilla was determined to be close to 99 % for a healthy hair follicle. Imaging of pilosebaceous units is useful for the diagnosis and understanding of pilosebaceous disorders such as alopecia and acne. This is also highly significant in the context of developing hair care products and hence might generate tremendous interest among multinational giants in the personal care products industry.

#### 2.2.2 Skin cancer

The number of skin cancer cases worldwide has risen noticeably in recent years. Skin cancer can be categorized into three main types; basal cell carcinoma, squamous cell carcinoma, and melanoma, in which the first two types are collectively called non-melanoma skin cancers and are seldom life threatening. Melanoma skin cancers are rare but they are potentially aggressive and usually start in pigmented lesions such as a mole or birthmark. Surgical intervention of these lesions is the gold standard for treatment which has evolved towards tissue sparing for better aesthetic results, namely, Mohs micrographic surgery (MMS) whereby the cancer is removed in layers until no cancer remains. Since MMS is time-consuming and requires special expertise and training, it can benefit from pre-operative 3D imaging of the skin cancer’s size and depth for exactly defined excision with improved clearance, lower relapse rates and shorter surgery time. Skin-directed imaging tools can also enhance the management of skin cancers when aggressive tumors are detected subclinically.

**Fig. 4:**
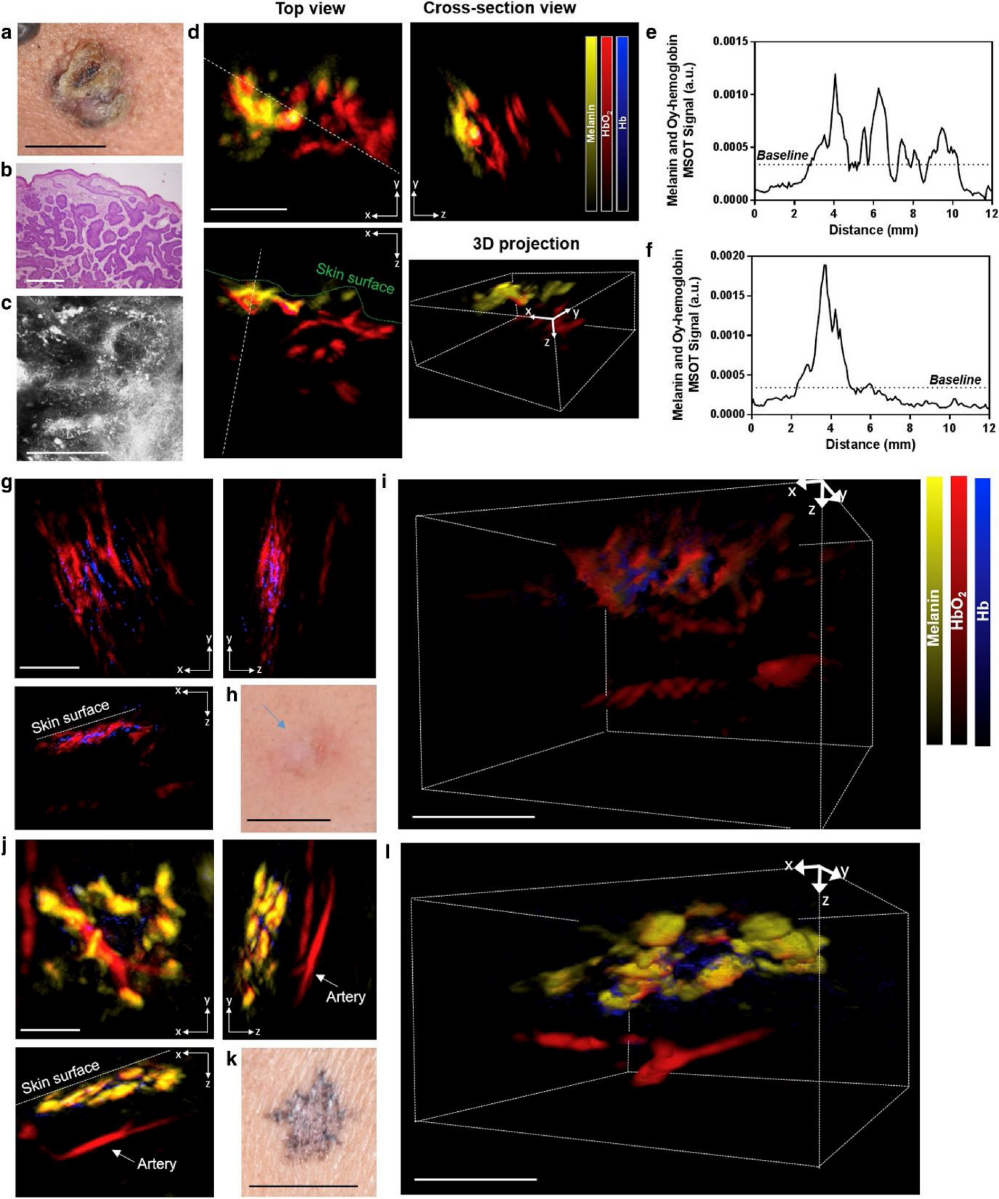
Clinical and PA images of non-melanoma skin cancer on representative patients. (a–c) Clinical, hematoxylin and eosin staining and reflectance confocal microscopy image of a basal cell carcinoma on a cheek. White scale bar =500 μm. (d) PAI orthogonal images and 3D projection of the lesion with the white lines drawn indicating the distance on which PA signals were to give tumor (e) length and (f) depth. PA images of a basal cell carcinoma on (g–i) a Caucasian patient with Fitzpatrick skin type II and an (j–l) Asian patient with Fitzpatrick skin type IV. Reprinted with permission from Ref. [82].

Label-free *in situ* PAI has been exploited to measure the size of cutaneous cancers due to the functional contrast of these lesions. Several studies using multispectral PAI configurations equipped with handheld transducer probes were demonstrated to measure the depth of *in situ* pigmented skin lesions which were validated with histological measurements after the lesions were excised or undergone MMS [[81], [82], [83], [84], [85], [86]]. The handheld implementation makes it flexible to access the skin cancer lesions which usually appear on sun-exposed areas such as the face and neck. As Chua et al. reported, when the MSOT 3D map of non-melanoma skin cancers were acquired, lesions margins could be visibly differentiated from the soft tissue in the vicinity due to the presence of melanin chromophore (Fig. 4). The length and the deepest infiltration dimensions of melanin signals were measured and they show a high correlation with the length and depth of histological resections of the tumors via a linear regressive fit (*r* = 0.9) [82]. Generally, lesion dimensions measured by PAI are overestimated compared to histological measurements obtained after excisional surgery, probably due to the dehydration of the tissue samples in histology preparation. Notably, most non-melanoma skin cancers appear pigmented in people of color while they appear erythematous on patients with lower Fitzpatrick phototypes. For example, erythematous lesions were shown as a mass of HbO_2_ signals with no melanin signals present (Fig. 4). Therefore, the spectral imaging ability of these systems in differentiating signatures of hemoglobin from melanin allow both pigmented and non-pigmented cutaneous lesions to be mapped with accurate lesion dimensions [82].

As mentioned earlier, melanoma skin cancers can spread throughout the body and the first site of metastasis is the nearby lymph nodes. Identification of metastasis in the lymph nodes is imperative in accessing the extent of the disease. Both *in vivo* and *ex vivo* studies examining the capability of multispectral PAI to detect melanoma metastasis in human lymph nodes have been reported [[87], [88], [89], [90]]. Stoffels et al. reported that MSOT imaging improved the accuracy of tumor metastasis rate in sentinel lymph node biopsies compared with the established standard protocols (22.9 % versus 14.2 %) [88]. To achieve *in vivo* imaging of the lymph nodes, the exogenous contrast agent ICG was administered peritumorally into patients with melanoma. The dye was visualized in the sentinel lymph node up to a depth of ∼10 mm beneath the skin with a 100 % agreement rate with the conventional radioactive tracing and pathological analysis methods.

These reported studies exhibit how PAI can be exploited to pre-operatively map the skin cancers to aid the surgeons in planning their excision surgery to save time and increase the accuracy of lesion margin removal. However, the challenges for PAI in skin cancer detection remain as the system’s detection limit of the imaging depth is only about 10 mm [82,83] and thus the technical design of the handheld probes have to be modified to address this pitfall. Furthermore, 3D mapping of the skin lesions are sensitive to motion so acquisition with artefacts have to be discarded. Future studies to validate the use of PAI in delineating skin cancer lesions would be using the pre-operatively determined tumor dimensions in the surgical planning process and judging the extent of cancer clearance.

#### 2.2.3 Inflammatory skin diseases

**Fig. 5:**
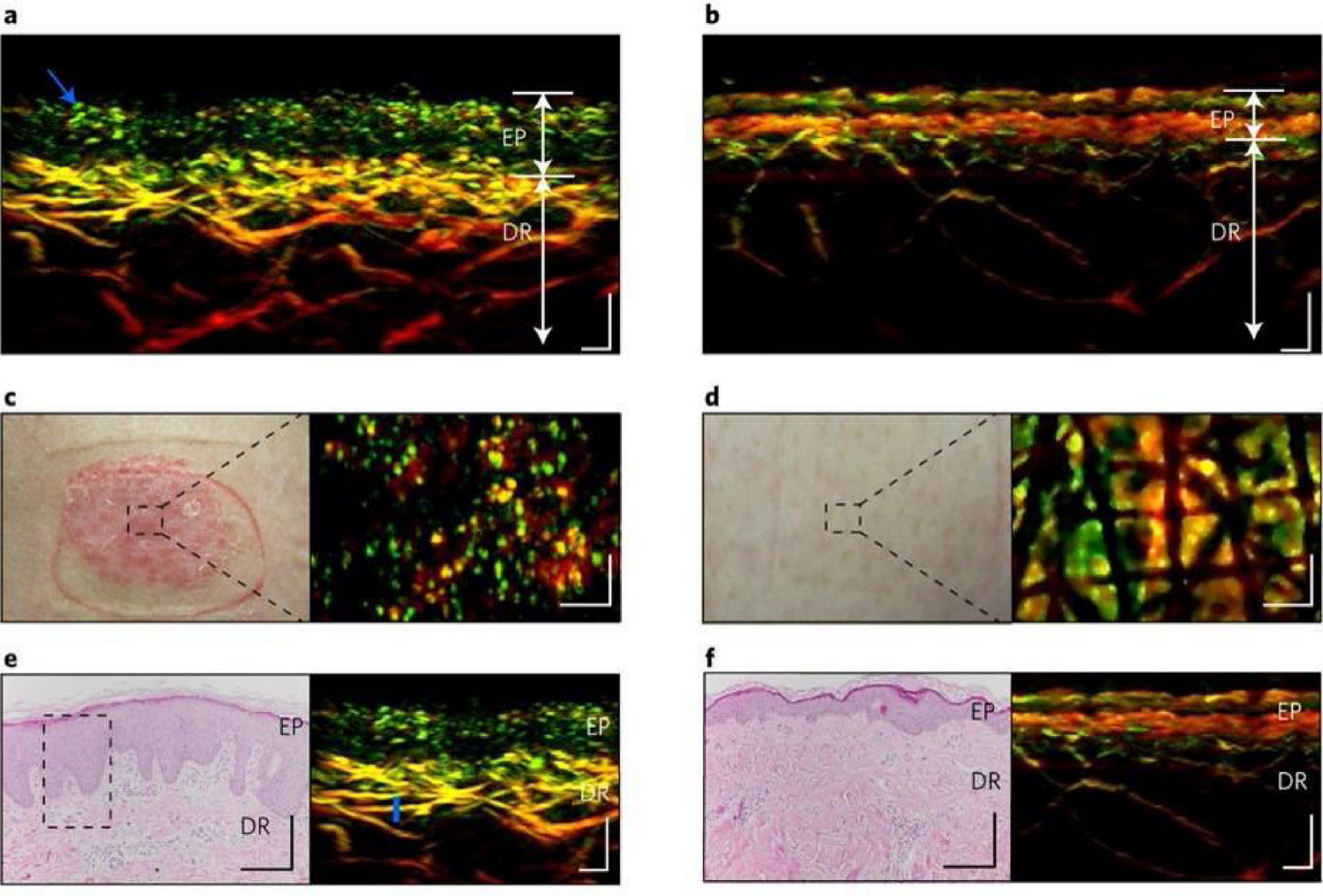
RSOM imaging of healthy skin and adjacent psoriatic lesion with histology validation. RSOM cross-sectional images, clinical images and histological images of (a, c, e) psoriatic skin and (b, d, f) adjacent healthy skin. Psoriatic skin exhibited elongated capillary loops near to the skin surface, thicker epidermis, absence of melanin and increased vascularization of the dermis which is validated by the histology images. Scale bars; 200 μm. Reprinted with permission from Ref. [91].

Inflammation is a systematic disease and can be manifested in macrovascular and microvascular disorders. While the former can be evaluated through sonography, pathological changes in peripheral small vasculatures can be visualized using PAI by virtue of the absorption of Hb and HbO_2_. Therefore, inflammatory systemic diseases manifested in dermatologic conditions such as psoriasis and eczema can be possibly imaged and monitored by PAI. Aguirre et al. reported the use of single wavelength RSOM in a pilot study to visualize skin architecture and vascular features in psoriatic skin, permitting the identification and quantification of inflammation landmarks [91]. Compared to healthy skin, psoriatic skin displayed increased epidermal thickness, elongated and dilated capillary loops and heightened dermal vascularization (larger diameter, denser in square area and more tortuous) which was corroborated with histological examination as shown in Fig. 5. From these objective measurements of the inflammation metrics, a calculated index of PA features were conceived which correlated well with the clinical Psoriasis Area Severity Index (PASI), the latter of which do not reflect the structural characteristics of the skin.

The vascular abnormalities seen in eczema patients are similar as reported by Zabihian et al. whereby a high-resolution PAM system integrated with optical coherence tomography (OCT) system was used [92]. Hypervascularization was observed in a subject with chronic hyperkeratotic hand eczema whereby the capillary loops were closer together and capillaries were present between the curves of the loops. In a subject with dyshidrotic hand eczema where scar tissue was existent, the vessels in the vascular plexus were narrower at the border between the scar tissue and healthy skin [92]. Evidently, inflamed skin lesions exhibit thickened epidermis and vascular patterns with dilated vessels arranged in a disordered network. The acanthosis of the skin lesions may cause high scattering within the tissues and may limit the imaging depth of OCT, diminishing the visibility of dermal structural features. Another inflammation indicator could potentially be the oxygenation status of the vessels measured via PAI as reported by Ishida et al. [93]. After a week post-treatment with ixekizumab of a patient with psoriasis on extremity digits, the oxygenation saturation level increased from 72 % to 89 % in the palmar digital veins. This increase in oxygenation was attributed to the lower oxygen consumption in the tissues as inflammation decreases, thus increasing the oxygenation saturation level in the vessels.

### 2.3 Vascular imaging in extremities

PAI’s label-free sensitivity to both HbO_2_ and Hb makes it an attractive modality to study and visualize vasculatures in all superficial organs. The different configurations of PAI systems have been utilized to grant in vivo structural and functional information (e.g. sO_2_) in human skin. Mesoscopic and microscopic configurations are suited for imaging cutaneous microvascular network down to individual vessels, as superficial vasculature can act as a site for surrogate markers of peripheral vascular diseases described below. Other modalities exploited for quantifying peripheral vasculature include magnetic resonance angiography (MRA) and MRI. However, these techniques require the use of external contrast agents and workup which may limit their clinical relevance. Laser speckle sensing has also been adopted to measure blood flow but being single-wavelength, it cannot quantify the tissue oxygenation level nor image the vasculature architecture. Meanwhile, the tomographic configuration of PAI in hand-held mode can be useful to image and interrogate specific larger and deeper blood vessels. Feasibility studies utilizing both volumetric 3D [48,94,95] and 2D array [96] hand-held probe designs to image human vasculature has been demonstrated. Taruttis et al. demonstrated the use of multispectral optoacoustic tomography (MSOT, iThera Medical GmbH) equipped with a handheld detector with a curved geometry (135° coverage) which was capable of penetrating 10 mm deep into the tissue to image major blood vessels and microvasculature [97]. Large blood vessels in the millimeter-scale such as tibialis posterior and dorsalis pedis arteries in the leg were visible from the contrast attributed to HbO_2_ signals. MSOT was also shown to resolve microvasculature of diameter 90−110 μm on the medial and distal big toe. The arterial pulse was also observed in real-time MSOT visualization as each laser pulse generated an image frame.

#### 2.3.1 Cutaneous microvasculature

The clinical studies described in this section were acquired on healthy subjects to judge the feasibility of PAI as an imaging tool to visualize microvasculature for potential disease applications. AR-PAM has been demonstrated to show the microvascular response on the palm to intermittent arterial occlusions by measuring the relative changes in oxygen saturation level and capillary perfusion [98]. A decrease in PA signals was observed during an occlusion after releasing which there was an increase, indicating a reduction and improvement in perfusion during and after ischemia respectively. The same change was reflected in the relative Hb concentration as well following occlusion and release. PAM imaging has also revealed that the microvasculature structure differs in between acral and non-acral areas [99]. Compared to the microvasculature in the palm, strong PA signals were observed in the epidermal–dermal junctions of the forearm. This was attributed to the denser network of perfused capillaries in the forearm, creating stronger and broader PA signals.

The mesoscopy configuration of PAI systems can penetrate beyond the optical diffusion limit and image over a bigger volumetric space in the skin. The ultra-wideband transducer in the RSOM system makes it capable of resolving the microvascular structure of the skin that includes small vessels from capillary loops near the epidermis-dermis junctions and bigger arterioles and venules in the vascular plexus – in a label-free mode [100]. The high frequency band during reconstruction revealed the capillary loops extending from the lower plexus, represented as ‘dot’-like structures. Capillary loops are situated below the junction whereby only the apex of the loops are visualized due to the directivity of the photoacoustic signals in the axial direction. From the low frequency band, the bigger vascular plexus forming the lower horizontal plexus can be visualized. The RSOM system when fitted with per-pulse tunable 100 Hz laser with a range of 460–650 nm is capable of spectral measurements to resolve the different main tissue absorbers i.e. melanin, Hb and HbO_2_ [77]. This particular setup is termed Multispectral Optoacoustic Mesoscopy (MSOM). This also granted additional spectroscopic data to obtain the sO_2_ values in single blood vessels, measuring about 85 ± 4 % in superficial vessels and about 54 ± 7 % in a draining vein in the dermis of the forearm. These values can be corroborated by other optical measurements such as reflectance spectroscopy and near-infrared spectroscopy of the bulk blood sO_2_ values of the forearm. The RSOM system has also been reported to resolve the microvasculature on the nailfold of healthy subjects, even in cases whereby the epidermis thickness is beyond the penetration depth of a standard clinical optical microscope [101]. Studying the nailfold microvasculature is useful to assess the early diagnosis of systemic sclerosis or scleroderma. The capillary density and maximal capillary diameter of the nailfold microvascular architecture were both quantified to derive objective parameters to aid clinicians in diagnosing systemic sclerosis, by observing avascular areas and atypically large capillaries. The quantified parameters as measured by RSOM was comparable to that measured by the optical microscope. Given RSOM’S capabilities to resolve the different layers in the skin morphology, the skin microvasculature response to local temperature rise could also be investigated [102]. Upon local heating of both volar and dorsal forearm, vascular density was observed with the RSOM signals in the deeper depths in the lower frequencies (bigger structures), signifying local vasodilation. Quantitation of the vasodilation revealed more than 1.7 times blood volume change and an increase in vessel diameter by 63 %.

PAI may also be valuable in the imaging of perforator vessels in anterolateral thigh flaps, usually used in the reconstruction surgery of the head and neck. In investigating this hypothesis, Tsuge et al. performed in vivo imaging of anterolateral thigh perforators and their furcate architecture in the subcutaneous tissue of healthy subjects, using a PAI system with a hemispherical detector array that acquires data in a spiral pattern within a plane [103]. The microvessels in the subcutaneous layer, particularly those in slanting or horizontal alignment, were visualized up to a depth of 13 mm while the PAI system demonstrated a limitation in visualizing subfascial vessels. Mapping of the vasculature in 3D revealed the branching architecture of perforators, suggesting the potential of PAI to be used as an intraoperative imaging modality during thin anterolateral thigh flap surgery.

These above-mentioned studies mainly exploited the endogenous tissue contrast to study the vascular architecture and blood oxygenation. In contrast, Lutzweiler et al. demonstrated PAI’s capability to measure the dynamic kinetics and clearance of an externally-administered contrast agent in a volunteer’s finger [104]. ICG was injected into superficial vein of the volunteer’s arm. A customized curved detection array with an angular coverage of 266° was utilized in the study to fit a finger and to acquire 10 cross-sectional images of the finger per second. Vasculature on the palmar side of the finger could be visualized through endogenous contrast and by monitoring the ICG PA signals in these vessels over time, ICG’s kinetic data could be fitted into a dual exponential function to derive the circulation and decay time of the contrast agent.

#### 2.3.2 Vascular dysfunction in diseases

PAI’s ability to resolve larger blood vessels and microvasculature makes it an attractive modality to investigate diseases whereby vascular dysfunction or remodeling is a marker for disease activity and progression. By studying the markers, an improved understanding of the diseases can be achieved to provide better management of the disease for the patients and healthcare providers, including improved disease stratification and more efficient assessments and follow-up. One such disease as mentioned above is systemic sclerosis. Systemic sclerosis is an autoimmune disease of the connective tissue, typified by skin and internal organs fibrosis and progressive vasculopathy. Masthoff et al. evaluated the extent of microvascular dysfunction in systemic sclerosis patients compared to healthy individuals with the use of MSOT equipped with a handheld detector with a center frequency of 4 MHz, offering 2D cross-sectional images with a FOV of 30 × 30 mm^2^ and 100 μm reconstructed pixel size [105]. In comparison to healthy individuals, the subcutaneous finger tissue of systemic sclerosis patients exhibited considerably lower HbO_2_ and HbT MSOT values (∼ 50 %). When systemic sclerosis patients of stable prognosis were compared to their counterparts with progressive prognosis, the latter exhibited lower Hb, HbO_2_ and HbT MSOT values. This proof-of-concept study provided evidence of inter-individual variation of the disease and how non-invasive longitudinal monitoring of these patients is essential for individualized treatment plans. Raynaud’s phenomenon is the most common manifestation of systemic sclerosis and it is characterized by spasms of arteries, thus impeding blood flow and it is exacerbated by stress or the cold. This medical condition has been similarly investigated via PAI by Eisenbrey et al. to enumerate tissue oxygenation in the nail bed and nailfold as an avenue to diagnose and assess Raynaud’s phenomenon [106]. PAI was performed on nail beds of patients with Raynaud’s associated systemic sclerosis along with healthy subjects using a Vevo 2100 LAZR system equipped with an LZ-250 probe in oxy-hemoglobin quantification mode at baseline and upon cold stimuli. While the number of vessels imaged in the FOV were the same between the disease and control groups, Raynaud’s patients exhibited a more distinct decrease in sO_2_ levels of the vessels upon cold stimuli with time compared to the healthy subjects. The authors maintained that measuring the oxygenation status of the vessels upon cold stimulus is more useful in the diagnosis of Reynaud’s phenomenon.

PAI has also been exploited to quantify imaging features for the diagnosis and management of vascular malformations [107]. Vascular malformation refers to congenital vascular abnormalities of a combination tangle of veins, arteries and lymph vessels or individually. MSOT imaging of venous malformations and arteriovenous malformations revealed that they exhibit higher HbO_2_:Hb ratios by approximately 40 % compared with healthy tissue with arteriovenous malformations exhibited significantly higher HbO_2_:Hb ratios compared with venous malformations. When 3 patients presented with arteriovenous malformations underwent embolization treatment (i.e. obstructing a blood vessel), the HbO_2_:Hb ratios decreased significantly but was still higher than that of healthy subjects. Unsurprisingly, partial embolization treatment only resulted in a conservative reduction in HbO_2_:Hb ratios. Venous malformations are however treated with drugs to shrink the malformations, i.e. percutaneous sclerotherapy. Similarly, the HbO_2_:Hb ratio of the venous malformations in 2 patients decreased upon successful sclerotherapy. This study signifies that PAI is a good modality in facilitating the intervention management of patients with vascular malformations.

#### 2.3.3 Wound imaging

Chronic wounds are wounds that exhibit little or no healing over extended periods of time. Although the precise mechanism behind the prevention of wound healing is unknown, it is thought that a local loss of oxygenation is one of the causes. Therefore, sO_2_ levels of the wound is potentially one of the parameters in studying chronic wounds. As multi-wavelength PAI is able to measure sO_2_ level, it can be exploited for wound imaging to provide label-free 3D information about the oxygenation at ultrasound resolutions. Petri et al. first demonstrated the use of PAI in assessing chronic wounds, in a study whereby 5 patients were imaged with PAI at baseline and post-application of topical hemoglobin spray onto their chronic leg ulcers [108]. Topical hemoglobin spray is a novel therapeutic intervention to increase the oxygenation in the ulcerated areas by performing as a transporter for ambient oxygen to the wound *in situ*. PAI imaging showed that the spray increased the oxygenation of the ulcers on the surface and in the deeper ulcerated tissue. In a subsequent study with 49 patients, about half of the patients were identified as non-responders to the hemoglobin spray therapy with a less than 10 % increase in oxygen saturation level, indicating that their leg ulcers were incapable of self-healing and therefore necessitate a removal of the sclerotic tissue by surgery [109]. This shows the potential of PAI as an adjunctive diagnostic tool with a treatment intervention in appraising chronic wounds and tailoring wound care management to the individual patient.

### 2.4 Carotid vessel imaging

Carotid artery disease is the most common form of heart disease, with stroke being the risk factor. Carotid duplex ultrasonography (DUS) is frequently employed in clinical practice to screen for any blockage or narrowing of the carotid arteries commonly caused by atherosclerotic plaques. DUS grants information on the plaque’s location, calcification presence and mobility characteristics without the use of any contrast agents or ionizing radiation. However, it is unable to dispense information on the plaque’s composition and structure. Because PAI bestows a comprehensive platform for structural and functional imaging, it is advantageous for cardiovascular imaging. Herein, we will concentrate on non-invasive PAI of the carotid artery [110]. The use of intravascular PA (IVPA) imaging, a catheter-based approach delivering a direct visualization of human coronary arteries [[111], [112], [113]] is beyond the scope of our review and will not be discussed herein. Its clinical use is still inundated with challenges and is for now restricted to visualizing ex vivo human carotid artery tissues. Efforts to emulate non-invasive PAI of diseased human carotid artery have been reported whereby human carotid the ex vivo artery tissue was embedded in a neck mimicking phantom and imaged [114].

In one of the first demonstrations of non-invasive PAI of the carotid artery, Dima et al. designed both a linear and curved ultrasound array probe for a PAI system tailor-made for carotid imaging [115]. The former array performed at 5–7 MHz frequency with 128 elements while the latter array was designed for central frequency of 5 MHz. The curved array demonstrated better imaging performance, giving a more thorough tomographic visualizations, with the vessels imaged showing superior likeness to the tissues. When the curved array was placed on the lower right neck of a healthy subject, the right common carotid was visualized, up to 18 mm under the surface of the skin. Nearby, the right internal jugular vein was identified along with the external jugular vein. The PAI images were corroborated by DUS which was acquired at the same location on the neck.

Merčep et al. undertook a hybrid MSOT, pulse-echo US and color Doppler imaging approach via a multi-segment detector array that simultaneously offers blood flow measurements and sO_2_ levels in the carotid artery region of a healthy subject [116,117]. Vascular structures appear in the MSOT images due to the hemoglobin contrast while they appear hyperechoic in the US images. Images acquired revealed a distinction between the sO_2_ levels of different vascular structures occupying at least 1−2 cm beneath the skin surface in the carotid space. As expected, the MSOT HbO_2_ signals were higher in the common carotid artery, while more Hb signals were present in the superficial microvasculature as well as the jugular vein. The blood flow of the common carotid artery was measured between the regular values of 30 and 40 cm/s as captured by color Doppler imaging. This multi-modal approach is advantageous as the complementary information imparted can aid in clinical decisions for an informed judgement of the carotid artery disease severity.

### 2.5 Musculoskeletal imaging

PAI’s capacity to provide high resolution optical contrast in soft tissues has been explored to map the morphology of cartilage, synovium, vascularity and bone tissue in human joints, particularly in inflammatory arthritis [118]. Inflammatory arthritis is a group of diseases whereby inflammation is present in the joints and often other tissues. Due to the inflammation, extreme hyperemia of the joint is observed from the hypervascularization and dilation of veins and capillaries present there. As the metabolic state of the inflamed tissue is apparent in its physiological states, i.e. blood volume, and blood oxygenation, PAI can be exploited to detect and study the inflamed tissue. A portable PA/US imaging system equipped with a hand-held linear probe emitting a single wavelength was first proposed by van den Berg et al. for the imaging of clinically evident synovitis in rheumatoid arthritis patients and control healthy subjects [119]. A superficial blood vessel was observed in both the inflamed and non-inflamed joints with more PA signals and more convergent PA features observed underneath the former. When the amplitude PA signals were quantified, the signals in the inflamed joints were 4 to 10-fold higher as compared to those from control groups. This feasibility study has demonstrated that PAI is sensitive to clinically evident synovitis in inflamed joints and should be further explored.

Jo et al. reported a PA and US dual-modality imaging system equipped with a linear probe and tunable dye laser to identify inflamed finger joints in patients and by quantifying the hyperemia and hypoxia associated with inflammation in their finger joints in relation to that of healthy subjects [120]. As the PA and US images were acquired simultaneously, the functional information of hyperemia obtained from PA could be co-registered with the US morphological tissue structures. The PA images revealed that the hyperemia present in the finger joints of the patients were visualized as a sheet or blob of blood while the hemoglobin signals observed were 66 times lower in the synovium of the normal subjects’ joints. Furthermore, the measured sO2 levels were lower in the arthritic joints in relation to the normal joints (0.582 ± 0.034 vs. 0.651 ± 0.020). This reported findings suggested that PAI, as a complement to US, is capable of investigating additional in vivo physiological biomarkers of inflammatory arthritis.

### 2.6 Gastrointestinal imaging

Under acute conditions of the clinical phenotypes of inflammatory bowel diseases i.e. Crohn’s disease (CD) and ulcerative colitis, the care management entails constant adjustment of therapeutic interventions to avoid severe irreversible complications. Like many other inflammatory disorders, CD also presents with poor correlation between intestinal inflammation with standard clinical scoring system and laboratory assessment, which demands for a more accurate objective assessment of the risk for relapses and associated complications. Existing colonoscopy based approach is time consuming and also associated with multiple levels of risk. In this context, hand-held MSOT system can be exploited to extract functional information in the colon as a rapid and noninvasive approach for the objective assessment of CD’s severity [121,122].

**Fig. 6:**
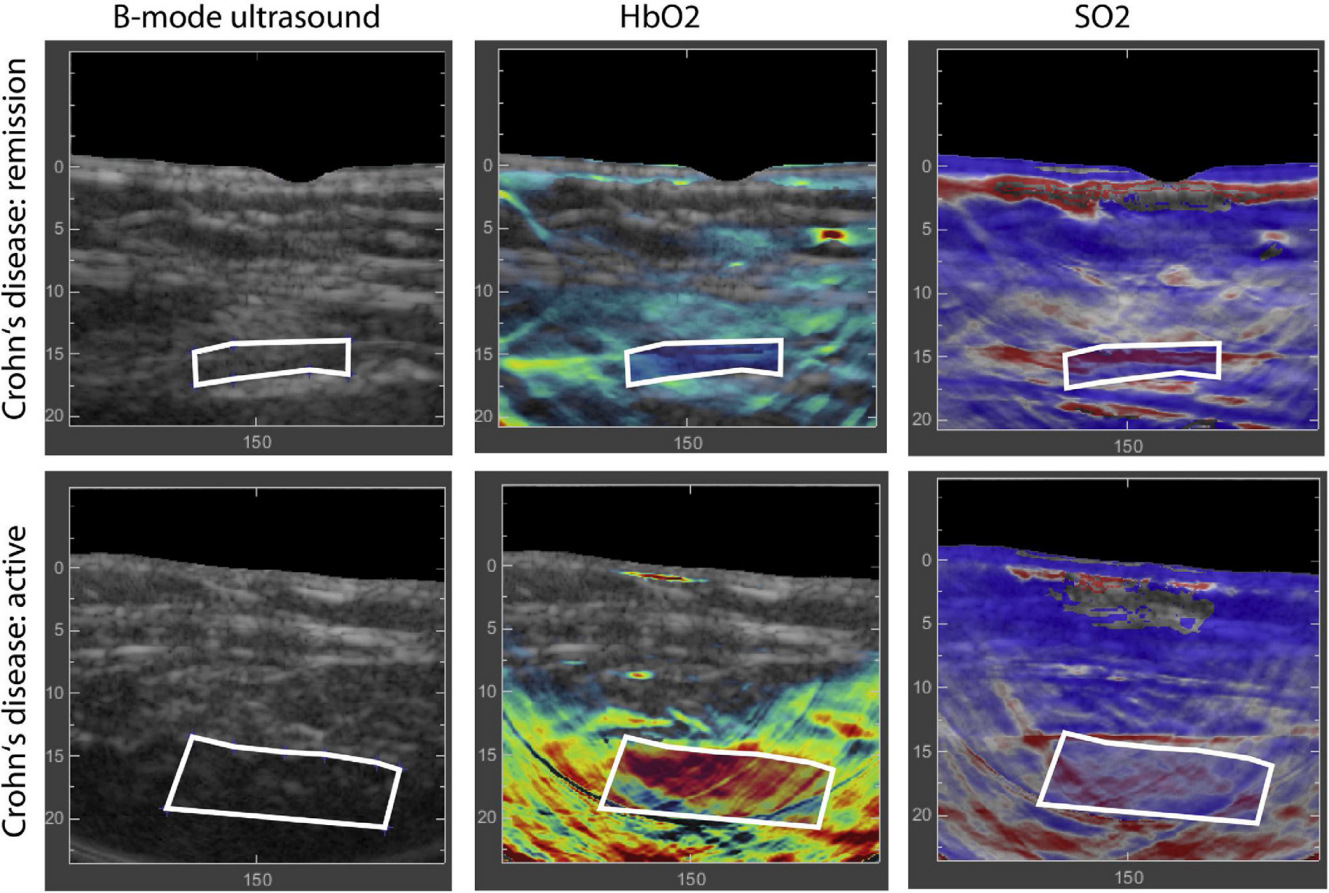
Representative MSOT images of the intestinal wall of a patient with Cohn’s disease (CD) in remission (upper panel) and a patient with active CD (lower panel). Identification of the intestinal wall was done using B-mode ultrasound image. It was observed that in contrast with the patient in remission, clinical and endoscopic CD activity was associated with increased signals for HbO_2_ and sO_2_ in the intestinal wall. Reprinted with permission from Ref. [122].

In a pioneering study conducted in 108 patients with varying degrees of CD severity, a clinical MSOT Acuity system fitted with a handheld detector (3−4 MHz, 256 transducer elements) acquired images from abdomen to image deeper organs and to monitor the changes in colon upon inflammation [121]. This study demonstrated a major distinction in single wavelength acoustic data at 760 nm and HbT signals between patients who show no inflammation in the intestines via scope and those with low grade inflammation. Furthermore, HbO_2_ and sO2 values increased progressively in correlation with disease severity as shown in Fig. 6 [122]. Quantified parameters from MSOT images were also compared between patients with active disease and those in remission, which was established from clinical scoring, endoscopic scoring and histologic data [121,122]. The capability of MSOT to spatially map the vascular Hb and HbO_2_ absorbers as markers for perfusion and inflammation in the intestinal walls with CD, offered functional information to objectively assess and to evaluate the level of inflammation in a reliable and noninvasive way.

### 2.7 Adipose tissue imaging

The critical role of adipose tissue in understanding metabolic disorders has recently been reported. There are two forms of adipose tissue: white adipose tissue (WAT) and brown adipose tissue (BAT). WAT acts as ‘reservoir’ for energy storage containing triglycerides, while BAT is specialized for thermogenesis to burn excess calories and produces heat mainly through the uncoupling reaction facilitated by uncoupling protein-1 (Ucp1). Over the last few years, there is a renewed spark in understanding these mechanisms and also the recently discovered “browning” process that involves cold induction or Ucp1 activation resulting in “brown-like” or “beige” adipocytes dispersed inside the major population of WAT but exhibiting genetic and biological characteristics of BAT, [123,124].

**Fig. 7:**
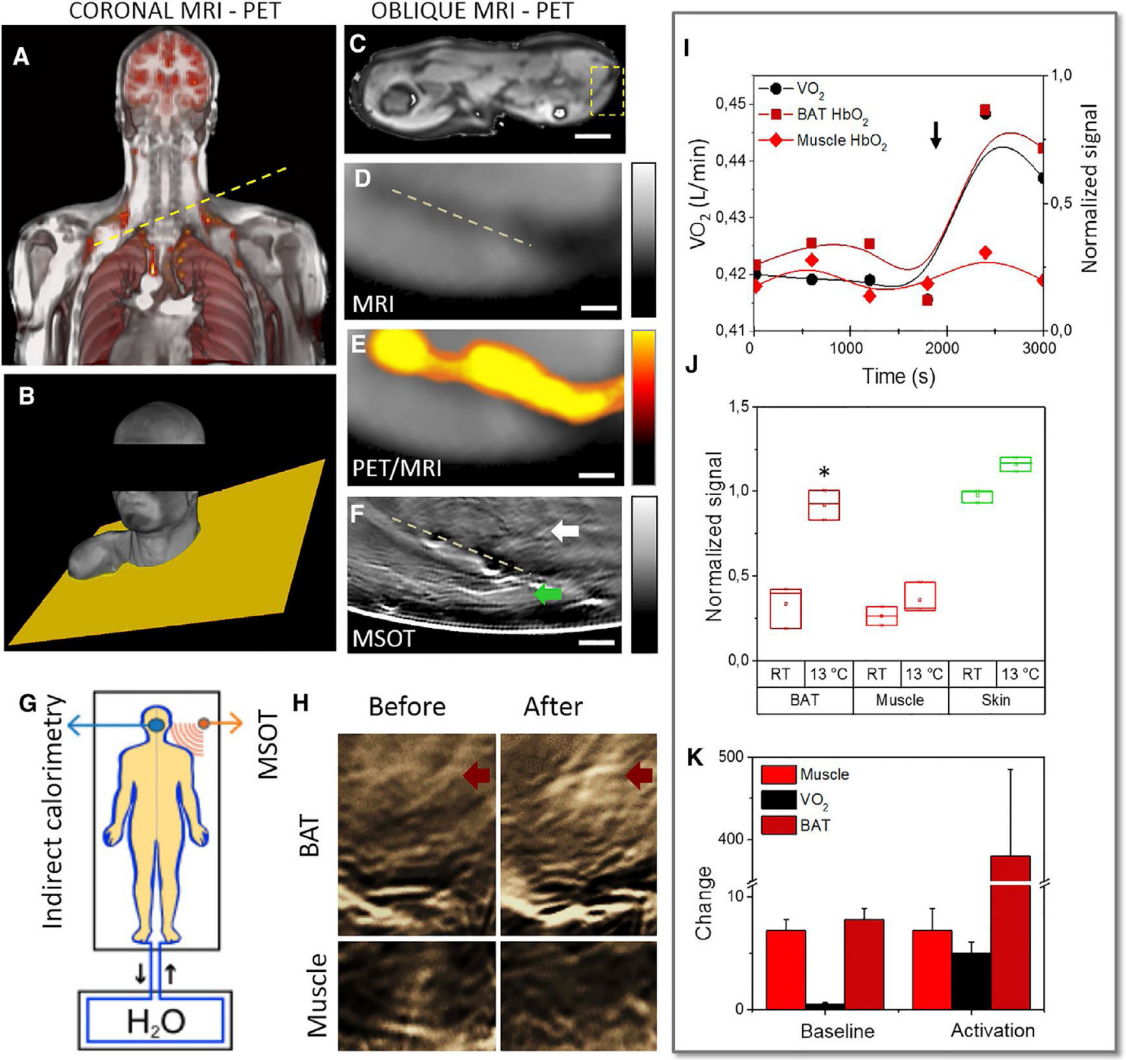
MSOT imaging of brown adipose tissue (BAT) of volunteers. (a–b) MR images and rendering of the upper torso of a subject-PET coronal co-registration of the upper torso of a volunteer; MR images of the (c) shoulder (d) supraclavicular regions of the volunteer with the yellow line and plane indicating where MSOT images were acquired. (e) Registered PET signal of 18F-FDG uptake in activated BAT on (d). (f) MSOT image of BAT and muscle from the same supraclavicular region. (g) Schematic of indirect calorimetry and MSOT measurements of cold activation of BAT using cold water. (h) MSOT images of BAT and muscle before and after cold activation from which the (i) HbO_2_ signals from BAT and muscle and corresponding oxygen consumption (VO_2_) values were measured. (j) Relative changes of signal intensity in BAT, muscle, and skin before and 10 min after cold exposure. (k) Comparative percentage change between baseline and baseline plus activation in muscle, VO_2_, and BAT ROIs. Scale bar; 5 cm in (c); 5 mm in (d–f). Reprinted with permission from Ref. [125].

Reber et al. utilized MSOT system fitted with hand-held 2D probe for noninvasive, label-free imaging of BAT in humans and also to resolve BAT activation based on hemoglobin gradients, which is in accordance with reported studies employing positron emission tomography (PET) [125]. Images from the BAT in the neck and the supraclavicular fossa of three subjects were acquired. Since the oblique resolution of MSOT system is higher than that of MRI and PET, a detailed view of the triangular muscle tissue could be observed just underneath the skin, corresponding to the other modalities, a seen as in Fig. 7. Upon BAT activation by cold exposure, the HbO_2_ signals in the BAT region increased considerably by 3-fold while no meaningful signal increase was observed in the muscle region (Fig. 7). The PA signal in the muscle exhibited a minor change of 1 % ± 2 % as compared to that of BAT region (372 % ± 105 %). The difference in lipid and TBV in the WAT and BAT were also observed in ten volunteers [125]. Resolving the spectral signatures of both regions revealed a precise difference in the MSOT spectra between BAT and WAT, with the former exhibiting 4-fold higher MSOT signal in the 700−900 nm region. Strong MSOT signal at 930 nm observed in both BAT and WAT region is due to the fat/lipids content which is confirmed by the absence of such signal in muscle. This study was instrumental in understanding the fundamentals of fat tissue metabolism and could be very well translated for understanding the role of adipose tissue in controlling obesity and diabetes. However, standardization of the MSOT approach with well-established MRI study protocol is necessary so that better image co-registration could be realized for more accurate comparison between water and lipid (fat fraction in MRI) signals.

In another study, Buehler et al. used a similar MSOT system with 2D hand-held probe to image subcutaneous lipomas (fatty tumors) [126]. They imaged six volunteers with lipomas located at different areas and correlated the results with clinical diagnostic US. MSOT images acquired at single wavelengths revealed better contrast in visualizing subcutaneous lipomas than that of US, particularly at wavelengths where fat exhibits minimum light absorption and hemoglobin absorbs the most. Spectral un-mixing showed that lipomas exhibit characteristic differences in signature between muscle tissues and normal tissues primarily due to the difference in absorption profiles of hemoglobin and lipid. This result is coherent with the fact that lipoma tissues are fatty and vascularized tissues while the primary absorbers in the muscle is blood and fat tissue *per se* contain less vessels than lipomas. The use of MSOT in conjunction with US for imaging lipomas can potentially enhance the diagnosis of subcutaneous soft tissue lesions due to the complementary images offered. Future studies could include developing MSOT imaging specific biomarkers based on spectral differences among other types of superficial soft tissue masses such as mesenchymal tumors, skin appendage, metastatic tumors (e.g. carcinoma, melanoma), tumor-like lesions (e.g. lymphoma, myxoma) and inflammatory lesions (cellulitis, abscesses).

## 3 Challenges and future directions

From this review, one can appreciate the extending list of organs and conditions that PAI helps to visualize. With breast cancer topping the list with CE marked Imagio™ and MSOT Acuity in markets for diagnosis, several novel clinical applications of PAI can be expected in the coming years with the advent of new custom proprietary PAI systems designed for clinical use. One such transpiring clinical application is in gynecology whereby multispectral PAI has been employed in pre-clinical studies to examine placental, fetal and maternal sO_2_ levels in normal and pathologic pregnancies in relation to pregnancy-related diseases [[127], [128], [129], [130]]. Recently, translation to the clinical stage was realized with a transvaginal fast-scanning photoacoustic endoscopy configuration which enabled high-resolution imaging of the microvasculature in the cervix down to capillary-size resolutions [131]. This system was tested in two pregnant subjects to observe the angiogenesis associated with cervical vascular remodeling during pregnancy. Parameters such as the microvessel density and total microvascular coverage area could be quantified from the images, with the former being the more reliable metric to quantify the extent of cervical remodeling. Having a transvaginal detector can potentially be used for *in vivo* ovarian imaging as well to detect and characterize ovarian cancer which have been demonstrated on *ex vivo* ovarian tissues previously [132,133]. However, several gaps need to be filled before such studies can be translated into clinical trials, including compliance to the laser safety limit, special design of transducer array to fit larger scanning areas, shortening of image acquisition time, development of large field-of-view imaging systems and achieving better contrast in the region of interest (ROI) compared to other regions of less interest. A full suite of contrast agents, those clinically approved for other optical imaging modalities [10] and totally new formulations [134] continually being tested for photoacoustic activity can offer advantage in enhancing the *in vivo* contrast of ROI.

Another area of potential clinical interrogation is to study neural activities and cognitive functions in the brain. While extensive pre-clinical brain studies using PAI have been reported, clinical translation of PAI brain imaging is challenged by the light and ultrasound attenuation through the thick human skull. This setback was addressed by Nie et al.who reported the first demonstration of transcranial PAI through an intact cadaver human skull by incorporating a photon recycler in the PAI system to reflect back-scattered light back to the skull [135]. Advances in wavefront shaping techniques which are known to significantly enhance the in situ optical flux and thus the signal to noise ratio are recently gaining importance in improving the penetration depth of photoacoustic imaging, particularly through turbid medium like the skull [[136], [137], [138], [139]]. This can potentially accelerate the clinical application of transcranial PA brain imaging, allowing the functional and pathological changes from diseases and different stimuli in the human brain to be observed.

While the clinical use of PAI has shown promising results in the pilot studies reported, multiple directions still exist for this imaging modality in its passage into clinical practice. Firstly, these studies are with a limited number of subjects, thus requiring larger cohort multicenter studies to establish the trends observed. Multicenter studies notably require high inter-operator and intra-operator reproducibility for the same PAI system utilized. While recent clinical studies have already started to evaluate these parameters [57,140], there is yet another aspect to seriously contemplate on. As many of these clinically tested PAI systems are prototypes from academic institutions and not of commercial quality yet, images from these systems considerably differ due to several factors such as laser power variability and image acquisition setup, thus requiring standardization across the spectrum of the systems. Efforts by Bohndiek et al. which are backed by volunteers from PAI community to successfully form a consortium termed International Photoacoustic Standardization Consortium (IPASC) to establish best-practice guidelines for image acquisition, analysis and reporting and a standardized validation for technical systems will largely help to verify the robustness of different PAI systems [141].

**Fig. 8:**
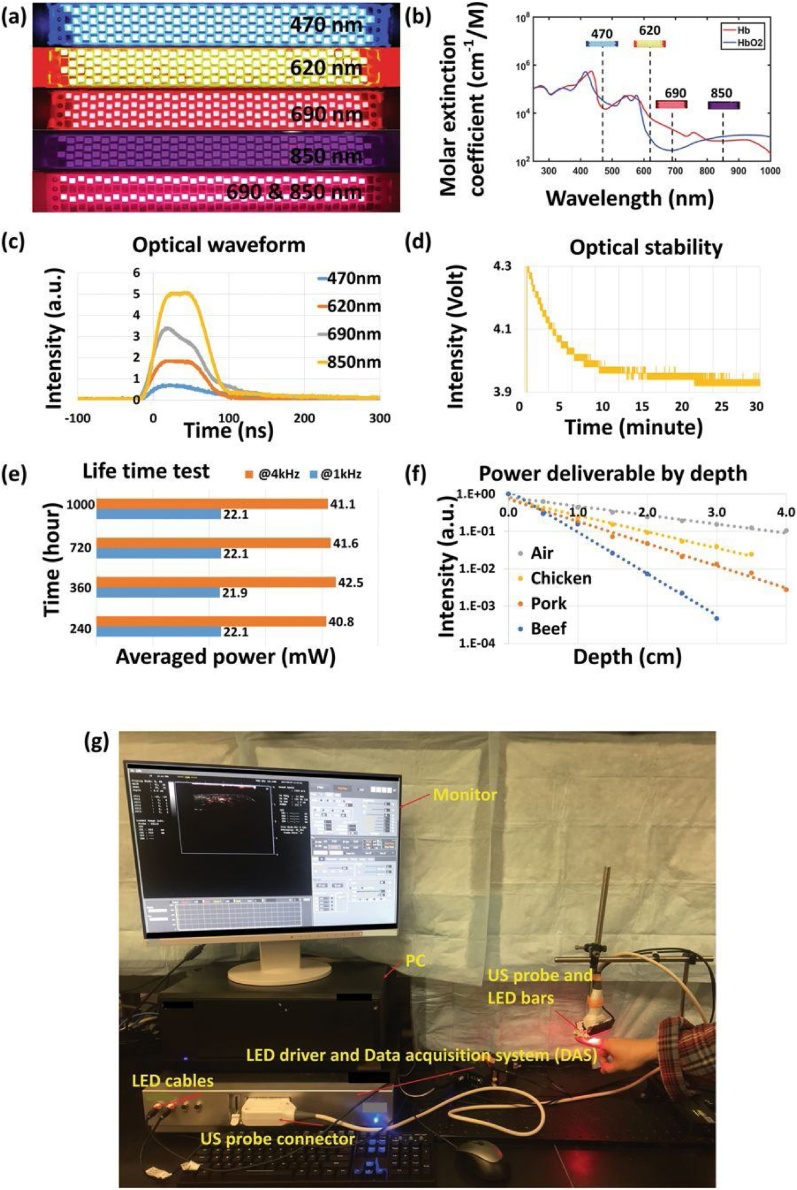
LED-based PAI system. (a–b) LED arrays working at different wavelengths in the spectral range of 400–900 nm. (c–d) Optical waveform and stability plots of LED emissions over time. (e–f) LED lifetime and deliverable power analysis at different time points and in different biological tissues, respectively. (g) Photograph of LED-based PA and US dual-modality imaging system. Reprinted with no changes from Ref. [143], image licensed under http://creativecommons.org/licenses/by/4.0/.

Secondly, adapting PAI systems to be portable and economical [142] is the key to gain widespread adoption in the clinics and such attempts are in the early development stage. Instead of utilizing expensive OPO or tunable dye lasers as the illumination source, low cost light emitting diodes (LED) are being investigated as a substitute laser illumination source in PAI systems [143]. AcousticX (Cyberdyne Inc., Tokyo, Japan) is one such commercial LED-based PAI system. The system utilizes LED illumination array from both sides of a regular ultrasound transducer, comprising of 4 rows of 36 single LED elements to illuminate an area of 50 × 7 mm^2^. To acquire multispectral data, each pair of LED array illuminates alternatively at 690 nm and 850 nm (Fig. 8). LED arrays working at other wavelengths of illumination at 470, 620, 690, 750, 810, 850, 930 and 980 nm have also been developed [143]. The system can achieve a lateral resolution between 0.55 and 0.59 mm and an axial resolution of 0.268 mm [144]. The advantages of the LED-based PAI setup include low cost, small footprint and negating the need for laser calibration and laser safety goggles. However, the larger pulse width and low power of LED limiting the efficiency of generated acoustic signals and penetration depth respectively have to be tackled to further improve their output energy before the LED-based PAI systems could achieve similar imaging quality and depth as conventional laser-based PAI systems. Moreover, the impossibility of tuning LED make them unsuitable for photoacoustic spectroscopic applications.

Another method to make PAI portable is by using Fabry–Perot film US sensors in the place of piezo transducers as demonstrated by Beard et al. [145,146]. A fully-optical PAI system consisting of a fibre-coupled laser as the illumination source and a Fabry–Perot sensor for acoustic detection has been demonstrated to image acoustically small structures volumetrically and at higher sensitivities, providing a spatial resolution in the range of 75−125 μm depending on the imaging depth and the FOV, which is typically 1−2 cm in both x and y axis. By being able to detect reliably the thermally induced peripheral vasoconstriction induced by thermal stimuli, it can be a prospective evaluation tool for patients with perivascular diseases By bringing several other significant advantages, such as high sensitivity, miniaturized sensor size, full transparency, wider response frequency, higher axial resolution and possibility of contact free measurements, fiber based ultrasound transducer opens up more opportunities in all kinds of PA endoscopy applications. Furthermore, clinical integration of PAI systems depends on a balance of functionality and seamless end-user operability. The systems have to be designed with the clinician or care provider as the final end-user in mind and such user involvement is vital for successful implementation of PAI in the clinics. As such, its tandem use with other conventional clinical imaging modalities such as MRI and OCT in a clinical workflow can facilitate this. Pre-clinical studies demonstrating multi-modal imaging of PAI with MRI [[147], [148], [149]] and OCT [150,151], fluorescence, two-photon and second-harmonic generation imaging systems [[152], [153], [154]] have been demonstrated in recent years, to exploit the complementary information granted from anatomical landmark imaging with high molecular and functional contrast delivered by PAI. As far as technology evolution goes, combining PAI with another optical imaging modality into one integrated system may not face as many setbacks as combining PAI with MRI for instance. Thus, it is important to identify the unmet clinical needs to be addressed by a multimodal integrated system as compared to sequentially performed PAI and MRI. Combining PAI and MRI would also require registration algorithms to align PAI molecular information with high-field MRI images based on mutual fiducial markers [149,155].

Thirdly, regulatory red tapes by regulatory authorities such as FDA in USA and CE Mark in European Union, present one of the challenges in translating PAI to the clinics. These regulations were put in place to assure quality, performance, and compliance of these technologies with American and European clinical standards. Nevertheless, a few commercial PAI systems are on their way to get regulatory approvals for clinical use, beginning with the first CE-marked PAI system in 2014, Imagio by Seno Medical Instruments, Inc. in Europe for diagnosing breast cancer. Other commercial PAI systems have followed suit such as MSOT Acuity (iThera Medical GmbH) with its CE Mark recently awarded for clinical use. Another present setback is clinicians having differing opinions on the value and effectiveness of PAI in the clinics, which can be mitigated by the active facilitation of the technology via an external national agency. The agency can act as a mediator across policy, systems and organizational levels which are important determinants in implementing a new technology in the clinics.

Finally, advancements in PAI systems has opened new doors in its applications pertaining to disease diagnosing, treatment, and monitoring, while generating copious amounts of data. Beyond just visual interpretation involved in clinical diagnostic paradigm, evaluations of such data can be enhanced by advanced computational analyses. While current systems come with relatively time consuming image re-construction and spectral un-mixing algorithms, further refining these to potentially reduce the processing time and improve the usability and image interpretability like in conventional sonography will impact the confidence level among clinicians to deduce reliable real-time functional information. Moreover, artificial intelligence (AI) can be utilized to make use of such generated data to translate them to clinically interpretable information. Acquired PA images could be deconstructed by AI into phenotypic descriptors to better quantify their visualizations. Such descriptors could characterize the shape, size, and pattern of the vascular remodeling in diseases, for instance. Deep learning, a subfield of AI, is an upcoming automatic approach that ‘learns’ phenotypic descriptors from a representative data to perform human-directed task‐specific applications and can outperform human capabilities. Clinically, the use of AI in tandem with PAI can potentially result in better disease management and patient outcomes by augmenting the qualitative assessments made by clinicians and introducing earlier interventions.

## Declaration of Competing Interest

Singapore Bioimaging Consortium has signed Research Collaboration Agreements free of financial interests with iThera Medical, GmbH and MicroPhotoAcoustics Inc. individually. VN is a shareholder in iThera-Medical GmbH, Munich, Germany.
